# Antimicrobial effectiveness of silver nanoparticles co-stabilized by the bioactive copolymer pluronic F68

**DOI:** 10.1186/1477-3155-10-43

**Published:** 2012-11-29

**Authors:** Carolina Alves dos Santos, Angela Faustino Jozala, Adalberto Pessoa Jr, Marcelo Martins Seckler

**Affiliations:** 1 Department of Chemical Engineering of the Polytechnic School, University of São Paulo (USP), São Paulo, Brazil; 2 Department of Biochemical and Pharmaceutical Technology, University of São Paulo (USP), São Paulo, Brazil

**Keywords:** Silver nanoparticles, Polymers, Pluronic™, Minimal inhibitory concentration, Gram-positive, Gram-negative

## Abstract

**Background:**

Silver nanoparticles (AgNps) have attracted much interest in biomedical engineering, since they have excellent antimicrobial properties. Therefore, AgNps have often been considered for incorporation into medical products for skin pathologies to reduce the risk of contamination. This study aims at evaluating the antimicrobial effectiveness of AgNps stabilized by pluronic™ F68 associated with other polymers such as polyvinyl alcohol (PVA) and polyvinylpyrrolidone (PVP).

**Methods:**

AgNps antimicrobial activity was evaluated using the minimum inhibitory concentration (MIC) method. The action spectrum was evaluated for different polymers associated with pluronic™ F68 against the gram negative bacteria *P. aeuroginosa* and *E. coli* and the gram positive bacteria *S. Aureus.*

**Results:**

AgNps stabilized with PVP or PVA and co-stabilized with pluronic™ F68 are effective against *E. coli* and *P. aeruginosa* microorganisms, with MIC values as low as 0.78% of the concentration of the original AgNps dispersion. The antimicrobial action against *S. aureus* is poor, with MIC values not lower than 25%.

**Conclusions:**

AgNps stabilized by different polymeric systems have shown improved antimicrobial activity against gram-negative microorganisms in comparison to unstabilized AgNps. Co-stabilization with the bioactive copolymer pluronic™ F68 has further enhanced the antimicrobial effectiveness against both microorganisms. A poor effectiveness has been found against the gram-positive *S. aureus* microorganism. Future assays are being delineated targeting possible therapeutic applications.

## Background

Contamination of medical products is frequently caused by the microbial adhesion of the patient’s glycoproteins, which deposit on the devices right after the implantation. Once the microbial adherence has occurred, the adhesion leads to the formation of a biofilm, which is resistant to most available therapeutic agents [1]. Therefore, improvement of a better strategy to prevent implant associated infections is an urgent need [2].

Silver nanoparticles (AgNps) have received a lot of attention for their antimicrobial activity because they are more effective in terms of minimum inhibitory concentration (MIC) than their ionic homologues (Ag^+^). Due to these antimicrobial properties, their incorporation into medical devices, tissues and other health related products promote higher preventive infection control [3,4].

It is believed that microorganisms are less likely to develop resistance to metals in comparison to conventional antimicrobials. This happens because metals in nanometric form act in several vital stages of bacteria and viruses metabolisms, so that consecutive mutations in the microorganism structure would be required for the development of resistance [5].

Although many earlier studies have demonstrated the antimicrobial activity of silver nanoparticles, the mechanisms involved are still not well understood. However, it has been hypothesized that the antibacterial activity of silver nanoparticles might be related to the crystallographic surface structure, the surface-to-volume ratio [6,7], the particle size [6-9], the particle shape [5] or the ionic forms of silver salts [10-12].

Silver nanoparticles stabilization plays an important role in their antimicrobial activity. For example, studies demonstrated that AgNps synthesized in the presence of different stabilizers (PVP, BSA and others) bind themselves to an HIV inhibitor peptide with different effectiveness [12]. Initially, it was believed that these polymeric systems used in drug delivery were biologically inert and that their functions came from the ability to (I) protect the drug/degradation active, (II) increase the half-life of these *in vivo* components, (III) facilitate their diffusion within the cell surface until the drug/active is released on the site of action. However, this paradigm has been modified due to recent evidences that these polymeric materials can drastically change the cellular response of microorganisms [13].

Pluronic™ copolymers are known to aid a number of therapeutic agents in their interaction with the human body. For example, when associated with antitumor agents, their concentration in tumor cells is increased, leading to a quick sensitization of tumors that show resistance against conventional treatments [13]. They are also capable of increasing the pharmacological activity of several antifungal and antibacterial agents against a large number of microorganisms [14-16]. It is likely that these characteristics of pluronic™ copolymers are caused by their ability to incorporate into the cell membrane and to translocate to the inside of the cells [13]. Inside the cells, pluronic™ may affect several cellular functions, such as the respiration process carried out by the mitochondrial system.

This study aims at evaluating the antimicrobial effectiveness of silver nanoparticles stabilized by pluronic™ F68 associated with other polymers such as polyvinyl alcohol (PVA) and polyvinylpyrrolidone (PVP). Pluronic™F68 was chosen in this study for its hydrophile-lipophile balance, which shows a more polar characteristic, a fact essential for its solubilization in aqueous medium during the AgNps synthesis.

## Results and discussion

A low antimicrobial activity against the *S. aureus* microorganism (MO) was found for AgNps irrespective of the stabilizing agents used (Table 1). For the PVP stabilized samples, either in the absence or presence of pluronic™ F68, such unfavorable inhibition of microorganism growth corresponded to a minimum inhibitory concentration (MIC) value of 50% of the concentration of the AgNps dispersion obtained in the synthesis process. When PVA was used as a stabilizer, a somewhat better MIC of 25% was observed; however, the use of pluronic™ F68 did not improve the antimicrobial effectiveness. An unfavorable MIC of 50% was also obtained for the AgNps stabilized with sodium citrate alone, while the addition of pluronic™ improved the MIC value to 25%.

**Table 1:** **Minimum Inhibitory Concentration (MIC) of polymer-stabilized silver nanoparticles against *S. aureus***

***S. aureus 10 6 CFU/mL Minimal Inhibitory Concentration***							
**wells**	**%**	**AgNps/PVP/pluronic pH 6.27**	**AgNps/PVA/pluronic pH 6.25**	**AgNps/citrate/pluronic pH 6.33**	**AgNps/citrate pH 6.1**	**AgNps/PVA pH 5.91**	**AgNps/PVP pH 5.97**
1	100	-	-	-	-	-	-
2	50	-	-	-	-	-	-
3	25	+	-	-	+	-	+
4	12.5	+	+	+	+	+	+
5	6.25	+	+	+	+	+	+
6	3.13	+	+	+	+	+	+
7	1.56	+	+	+	+	+	+
8	0.78	+	+	+	+	+	+
9	0.39	+	+	+	+	+	+
10	0.20	+	+	+	+	+	+
11	0.10	-	-	-	-	-	-
12	-	+	+	+	+	+	+

(+)Showed growth of microorganism. (-) No growth of microorganisms. 11 negative control/12 positive control.

A high antimicrobial effectiveness against the *E.coli* MO of AgNps was found for all of the studied stabilizing polymers (Table 2). When PVP was used as stabilizer a MIC of 3.13% was obtained and in the presence of the pluronic™ F68 co-stabilizer the inhibition value found was 1.56%. When PVA was used, 3.13% was obtained and when pluronic™ was added with PVA the value decreased even further to 0.78%, showing an improvement in the antimicrobial activity attributed to pluronic™ F68. In the samples stabilized with sodium citrate either alone or in association with pluronic™ F68, MIC values found corresponded to 1.56% of the initial concentration.

**Table 2:** **Minimum Inhibitory Concentration (MIC) of polymer-stabilized silver nanoparticles against *E. coli***

***E. coli 10 6 CFU/mL Minimal Inhibitory Concentration***							
**wells**	**%**	**AgNp/PVP/pluronic pH 6.27**	**AgNps/PVA/pluronic pH 6.25**	**AgNps/citrate/pluronic pH 6.33**	**AgNps/citrate pH 6.1**	**AgNps/PVA pH 5.91**	**AgNps/PVP pH 5.97**
1	100	-	-	-	-	-	-
2	50	-	-	-	-	-	-
3	25	-	-	-	-	-	-
4	12.5	-	-	-	-	-	-
5	6.25	-	-	-	-	-	-
6	3.13	-	-	-	-	-	-
7	1.56	-	-	-	-	+	+
8	0.78	+	-	+	+	+	+
9	0.39	+	+	+	+	+	+
10	0.20	+	+	+	+	+	+
11	0.10	-	-	-	-	-	-
12	-	+	+	+	+	+	+

(+)Showed growth of microorganism. (-) No growth of microorganisms. 11 negative control/12 positive control.

The antimicrobial activity of the studied AgNps against the *P. aeruginosa* MO depended on the stabilizing agents used, but in all cases improved when pluronic™ F68 was added as a co-stabilizer (Table 3). For PVP-stabilized AgNps the efficacy was low, with MIC values of 50% of the initial sample concentration. However, the effectiveness improved to a MIC of 12.5% in the presence of the pluronic™ F68 co-stabilizer. The use of PVA showed a MIC of 50% of the initial sample concentration and an improvement to 6.25% in the presence of pluronic™ F68. When sodium citrate alone was used, a MIC of 25% was found, whereas in the presence of pluronic™ F68 the MIC value improved to 12.5%.

**Table 3:** **Minimum Inhibitory Concentration (MIC) of polymer-stabilized silver nanoparticles against *P.aeruginosa***

***P. aeruginosa 10 6 CFU/mL Minimal Inhibitory Concentration***							
**wells**	**%**	**AgNp/PVP/pluronic pH 6.27**	**AgNps/PVA/pluronic pH 6.25**	**AgNps/citrate/pluronic pH 6.33**	**AgNps/citrate pH 6.1**	**AgNps/PVA pH 5.91**	**AgNps/PVP pH 5.97**
1	100	-	-	-	-	-	-
2	50	-	-	-	-	-	-
3	25	-	-	-	-	+	+
4	12.5	-	-	-	+	+	+
5	6.25	+	-	+	+	+	+
6	3.13	+	+	+	+	+	+
7	1.56	+	+	+	+	+	+
8	0.78	+	+	+	+	+	+
9	0.39	+	+	+	+	+	+
10	0.20	+	+	+	+	+	+
11	0.10	-	-	-	-	-	-
12	-	+	+	+	+	+	+

(+)Showed growth of microorganism. (-) No growth of microorganisms. 11 negative control/12 positive control.

The antimicrobial activity of solutions without AgNps were also investigated. It was found that aqueous solutions containing either PVA, PVP, pluronic™ F68 or sodium citrate were not active against microorganisms growth (data not shown). Therefore, the antimicrobial effectiveness of each formulation containing AgNps is attributable to the association of AgNps with the respective stabilizer and/or the co-stabilizer.

It is noteworthy that AgNps are more effective against gram-negative microorganisms, the *E. coli* and the *P. aeruginosa*. It is also against these strains that the pluronic™ F68 incorporation is more effective. These results are consistent with our previous work, in which pluronic™ F68 improved the antimicrobial effectiveness of the ceftazidime drug, a third generation cephalosporin with wide action spectrum, against *E. coli* and *P. aeruginosa* microorganisms [17].

The use of AgNps co-stabilized by pluronic™ F68 could aid in the decrease of the resistance phenomena [18] where the use of AgNps for burns treatment presented the onset of resistance against *Enterobacteriaceas, P.aeruginosa, Salmonella* and others.

The silver antimicrobial effectiveness is increased when incorporated in the copolymer, in comparison to pure silver nitrate. It was suggested that such phenomenon may be related to silver inactivation by nutrient broth or by the microorganisms themselves [19].

The silver antimicrobial effectiveness has been acknowledged for ages. Over the last few years, the use of silver or silver salts as key components to control the microbial proliferation has become increasingly popular. They are being currently incorporated in a wide variety of materials used in our daily lives, which range from the textile and hospital areas to materials used in personal hygiene, such as deodorants and toothbrushes [19-22]. A recent application is based on matrixes formed by collagen and bayberry tannin for the immobilization of silver nanoparticles (AgNps) [23].

In order to determine the susceptibility of microorganisms against AgNps, some methods reported the importance of standardizing MIC and other studies for AgNps evaluation when they are incorporated to medical and hospital products. Another important fact is that the presence of silver ions, resulting from the AgNps synthesis process, also has bactericidal potential [4,18]. For such reasons, these studies defined that the MIC should be determined by the percentage concentration (%) of the solutions obtained in each process. these recommendations make it possible to demonstrate the importance of stabilizing and co-stabilizing polymers to obtain more effective particles against different microorganisms, as well as the evaluation of the susceptibility of an unpurified solution composed by AgNps and Ag ions mixture.

AgNps and Ag ions accumulate in *E. coli* cells protein envelope and both AgNps and Ag ions seem to act similarly inside the cell, though their effectiveness is of nanomolar and micromolar order of magnitude for AgNps and Ag ions, respectively [4].

Amphiphilic polymers such as the ones from the pluronic™ class are highly relevant materials for the development of nanoparticles for medical use. Nanoparticles co-stabilization with pluronic™ did not show considerable modifications in particle size. The pluronic™ addition showed improvement also on colloidal stability and on the nanoparticles analytic characteristic of metals demonstrated through Raman [24].

## Conclusion

AgNps stabilized by different polymeric systems have shown improved antimicrobial activity against the gram-negative microorganisms *E. coli* and *P. aeruginosa* in comparison to unstabilized AgNps. Co-stabilization with the bioactive copolymer pluronic™ F68 has further enhanced the antimicrobial effectiveness against both microorganisms. A poor effectiveness has been found against the gram-positive *S. aureus* microorganism*.*

## Materials and methods

### Silver nanoparticles synthesis

The Turkevich method for metals nanoparticle synthesis has been adapted by the authors to deliver AgNps with good antimicrobial properties [25]. The method only involves benign substances, so it is suitable for applications in the human body. This method was applied here with further modifications to allow incorporation of the polymeric stabilizers and the pluronic™ co-stabilizer. A 500 mL jacketed reactor was completely filled with deionized water and heated to 89°C. Subsequently 90 mg of silver nitrate (AgNO_3_) and 135 mg of either PVA or PVP polymer were added (method proposed by Wang et al., 2004). Then 10 mL of a 1 wt% aqueous solution of sodium citrate was added to the reactor using a peristaltic pump, so that a molar ratio of silver nitrate to sodium citrate of 1:0.68 resulted. The chemical reduction of silver ions by citrate was allowed to proceed under controlled temperature and stirring. After 20 min of reaction, the reaction was stopped by cooling to ambient temperature. In some experiments, after 20 min of reaction 6 g of the pluronic™ F68 copolymer (molecular weight 8,400) was added, after which the reactor was cooled to 52°C and kept at this temperature under stirring for additional 20 min.

The amount of pluronic™ F68 added to the reaction was determined according to the critical micelle concentration/temperature. The pluronic™ mass added considers the formation of micelles of sizes of up to 100nm. If micelles are not formed, free monomers in the reaction medium promote the formation of particles over 300nm, i.e. outside the proper size range in this study.

The pH values of the AgNps dispersions were determined by a pHmeter (Accumet AR 20, *Fisher Scientific*, USA) at room temperature (25°C). The pH values in all preparations in this work were within the narrow range of 5.9 to 6.3.

### Bacterial strains

The microorganisms used were *Escherichia coli ATCC 25922*, *Pseudomonas aeruginosa* ATCC 9721 and *Staphylococcus aureus* ATCC 10390. These microorganisms were chosen following the recommendations of a Committee of the Biological Section of the Pharmaceutical Manufacturer’s Association and also because they are very common in hospitals. The microorganisms growth was carried out in 250 mL Erlenmeyer flasks, incubated in a rotational shaker at 100 rpm and 37°C for 24 h, using 50 mL of *Tryptic Soy Broth* (TSB). All cultivation media were prepared using distilled water and were autoclaved at 121°C for 30 min.

### Minimum inhibitory concentration

AgNps antimicrobial activity was evaluated using the minimum inhibitory concentration (MIC) method. The antimicrobial effectiveness was determined against the final microorganisms concentration of 10^6^CFU/mL (USP <51>). The action spectrum makes it possible to ensure the activity of AgNps stabilized by the different polymers and of the co-stabilizer pluronic™ F68. The minimum inhibitory concentration is expressed as the percentage of the original AgNps dispersion in the synthesis reaction medium (a MIC value of 25% indicates a solution containing 25% of the AgNPs present in the original synthesis dispersion). All samples were made in triplicates.

**Figure 1:**
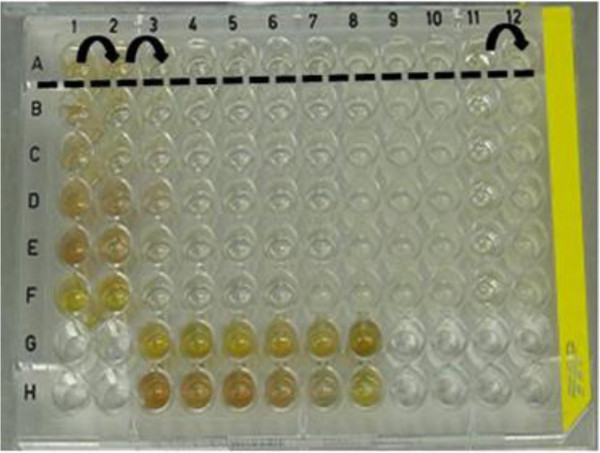
Minimum Inhibitory Concentration Methodology (MIC).

The MIC classic methodology is schematically shown in Figure 1[26] and was adapted to 96-well microplates. 300 μL of deionized sterile water were added on the exterior perimeter wells of the microplates to minimize the evaporation of the culture medium of the test wells during the incubation.

100 μL of the culture medium were distributed in 12 of the 96 wells, previously sterile, with the exception of well #1. In the first and second wells of the series, 100 μL of the AgNps dispersion was added. Both wells were homogenized with the aid of micropipette and 100 μL was transferred from well #2 to well #3. The transfer was successively repeated until well #11.

10 μL of the inoculum (microorganism tested) in known concentration, stipulated at 10^6^ CFU/mL, were added in all wells, with the exception of well #11. The plate was covered and was incubated at the optimum growth temperature for the microorganism for 24 h.

Afterwards, the results were studied, inoculating the content (5 μL) of each well in Petri dishes containing agar from the specific culture medium tested. The well with higher dilution was the MIC, in which the absence of bacterial growth was observed. Wells #11 and #12 were the positivo (Culture medium + inoculum) and negative controls (Culture medium + antimicrobial), respectively [26].

The same procedure was carried out for the silver nitrate for polymers PVA, PVP and pluronic™ F68, individually, in order to evaluate if they did not present any inhibitive effect on the MO.

## Abbreviations

AgNps: Silver nanoparticles; PVA: Polyvinyl alcohol; PVP: Polyvinylpyrrolidone; MIC: Minimum inhibitory concentration; AgNO_3_: Silver nitrate; MO: Microorganisms; TSB: Tryptic Soy Broth; CFU: Colony forming units; Ag: Silver.

## Competing interests

The authors declare that they have no competing interests.

## Authors’ contributions

CAS carried out the Silver Nanoparticles Synthesis. AFJ carried out the MIC assays. APJ and MMS were scientific advisors and provided essential advice and edited the manuscript. All authors read and approved the final manuscript.
